# Complete Genome Sequence of Gordonia polyisoprenivorans 135, a Promising Degrader of Aromatic Compounds

**DOI:** 10.1128/mra.00058-23

**Published:** 2023-03-14

**Authors:** Ekaterina Frantsuzova, Viktor Solomentsev, Anna Vetrova, Vasili Travkin, Inna Solyanikova, Yanina Delegan

**Affiliations:** a Institute of Biochemistry and Physiology of Microorganisms, Federal Research Center “Pushchino Scientific Center for Biological Research of Russian Academy of Sciences” (FRC PSCBR RAS), Pushchino, Moscow Region, Russia; b State Research Center for Applied Microbiology and Biotechnology, Obolensk, Moscow Region, Russia; c Belgorod State University, Belgorod, Russian Federation; University of Southern California

## Abstract

Gordonia polyisoprenivorans 135 is a promising degrader of aromatic hydrocarbons. It can utilize phenanthrene, anthracene, benzoate, chlorobenzoates, and phenol. The genome of strain 135 was completely sequenced; it consists of a single 5,988,360-bp circular chromosome (GC content of 67.01%).

## ANNOUNCEMENT

Polycyclic aromatic hydrocarbons (PAHs) are chemically stable carcinogenic and mutagenic compounds of crude oil (1). The ability to utilize PAHs is characteristic for many *Actinobacteria* members, but not many strains of the genus *Gordonia* are currently known to possess this property. A description of the genetic organization of the PAH catabolism pathway is presented in the work of Lin et al. (2).

Gordonia polyisoprenivorans strain 135 (previously Rhodococcus rhodnii 135) was isolated in 1998 from soil contaminated with oil, diesel fuel, and chlorophenols (latitude, 53°10′39′′N; longitude, 50°04′44′′E; Samara, Russia) using the enrichment culture method (3, 4) on Evans medium (5) with *p*-hydroxybenzoate as the sole source of carbon and energy. The strain is capable of utilizing phenanthrene, anthracene (6), benzoate (7), chlorobenzoates (8), and phenol (4). For long-term storage, the strain was kept in glycerol (40%) stocks at −70°С. For short-term maintenance, the strain was cultured on a Lysogeny broth agar plate at 27°C.

Genomic DNA was isolated from a fresh culture biomass (a colony) of Gordonia polyisoprenivorans 135 grown on LB agar using a DNeasy blood and tissue kit (Qiagen; 69506). Sequencing was performed using a MinION sequencer with flow cell FLO-MIN106 (Oxford Nanopore Technologies [ONT]). A library was prepared with a ligation kit (SQK-LSK109). Guppy 3.2.4 was used for base calling, which yielded a total of 1,335.8 Mb distributed in 332,510 reads with a Q of >10 (*N*_50_ is 14,599 bp).

Additionally, the same DNA sample was sequenced with an MGI platform (DNBSEQ-G400) using the DNBSEQ-G400RS high-throughput sequencing set (FCL PE150) (2 × 150 bp). A paired-end library was prepared with the MGIEasy universal DNA library prep set. We obtained 8,939,552 paired-end reads of <150 bp. The MGI and Nanopore reads were used for hybrid assembly with SPAdes 3.15.4 (9). The Nanopore reads with a length of >2,000 bp were assembled using Flye 2.9.1 (10), and a single circular contig was obtained. Next, SPAdes contigs were combined into replicons in SnapGene 6.1 (from Dotmatics; available at http://snapgene.com) using Flye data as the reference. The MGI reads were used to correct Nanopore or assembly errors using Bowtie 2 2.3.5.1 (11) and Pilon 1.24 (12) software. Default parameters were used for all software.

The Gordonia polyisoprenivorans 135 genome consists of a single 5,988,360-bp circular chromosome (GC content of 67.01%). Chromosome circularization was specified by ends overlapping. The average nucleotide identity (ANI) value was calculated using the EzBioCloud ANI calculator (13). DNA-DNA hybridization (DDH) was calculated using the Genome-to-Genome Distance Calculator 3.0 (GGDC) (14). The ANI value with the type strain Gordonia polyisoprenivorans NBRC16320 (BAEI00000000.1) was 98.68%, and the DDH value was 88.40%.

The *G. polyisoprenivorans* 135 genome was annotated with the NCBI Prokaryotic Genome Annotation Pipeline (PGAP) 4.6 (15). The chromosome contained 5,168 coding sequences, 3 rRNA clusters (5S, 16S, and 23S), 49 tRNAs, and 3 noncoding RNAs (ncRNAs). The genes of the PAH catabolic pathway were found in the genome of the strain 135, but their order was different from that of the strain *Gordonia* sp. CC-NAPH129-6 (2). The genome sequence data of Gordonia polyisoprenivorans 135 will enhance our understanding of the metabolism of PAH-degrading *Gordonia* strains.

The antiSMASH 6.0 (16) search for secondary metabolite clusters found 15 clusters on the chromosome, including clusters of ε-poly-l-lysine, ectoine, and aminopolycarboxylic-acid production.

### Data availability.

This genome project has been deposited at GenBank under the accession number CP116236. BioSample number SAMN32738803, and BioProject number PRJNA923796. SRA accession numbers were SRX19143368 for Oxford Nanopore data and SRX19144143 for DNBSEQ data.
